# Editorial: Medical overuse and underuse in healthcare systems

**DOI:** 10.3389/fpubh.2025.1590328

**Published:** 2025-04-15

**Authors:** Morteza Arab-Zozani, Loai Albarqouni

**Affiliations:** ^1^Social Determinants of Health Research Center, Birjand University of Medical Sciences, Birjand, Iran; ^2^Institute of Evidence-Based Healthcare, Faculty of Health Sciences and Medicine, Bond University, Gold Coast, QLD, Australia

**Keywords:** medical overuse, medical underuse, low-value care, unnecessary health care, healthcare system

The healthcare landscape is rapidly evolving with advances in medical technology, pharmaceuticals, and diagnostic tools (1). While these innovations have markedly improved patient outcomes and saved countless lives, they have also led to a concerning issue: medical overuse. Medical overuse refers to services that are more harmful than beneficial, do not enhance the quality and quantity of life, impose excessive costs on patients and the healthcare system, and are of low quality. If patients were fully informed, they would likely not request these services (2). This phenomenon, characterized by unnecessary or excessive medical interventions, poses significant risks to both individual patients and the broader healthcare system, in addition to imposing substantial costs (3). Addressing this growing concern requires education, policy reform, and systemic change.

The root causes of medical overuse are complex and include financial incentives, defensive medicine practices, patient demand, and a lack of clear clinical guidelines. The consequences are far-reaching. On an individual level, patients may suffer adverse effects from unnecessary treatments, experience avoidable discomfort, and face financial burdens due to high healthcare costs. At a systemic level, medical overuse contributes to rising healthcare expenditures, diverts resources away from essential services and potentially exacerbates health disparities (4).

One of the most pressing issues associated with medical overuse is its impact on healthcare expenditure. In many countries, healthcare spending continues to outpace economic growth, with overuse being a significant contributor. Unnecessary tests and treatments strain public budgets and increase out-of-pocket expenses for patients, leading to financial distress and even bankruptcy in some cases. Additionally, allocating resources to low-value care detracts from investments in preventive care, mental health services, and other critical areas that could yield greater population health benefits (5).

Beyond financial implications, medical overuse can directly harm patients. For instance, exposure to radiation from unnecessary imaging studies increases cancer risk, while inappropriate antibiotic use fuels the global crisis of antimicrobial resistance. Furthermore, overly aggressive treatment regimens can lead to complications, prolonged recovery times, and diminished quality of life. These adverse effects underscore the importance of balancing intervention with prudence, ensuring that medical decisions prioritize patient wellbeing above all else (6).

Several factors contribute to the prevalence of medical overuse and are among the drivers of medical overuse, the most important of which include financial incentives, through fee-for-service payment models, that encourage providers to order more tests and procedures because they are reimbursed based on volume rather than value. Another driver is defensive medicine, which, through fear of malpractice lawsuits, forces physicians to practice “defensive medicine” and prescribe more tests or procedures to protect themselves from potential lawsuits. Patient expectations are also an important driver because patients often expect certain interventions, even when evidence suggests that the interventions are unnecessary. Providers may be pressured to maintain patient satisfaction. Also, despite their important role, the lack of clinical guidelines is another driver. Inconsistent or outdated clinical guidelines can leave clinicians uncertain about the appropriate course of action and contribute to variation in care (7).

Several studies have shown that to combat medical overuse, coordinated efforts must be made at different levels of the healthcare ecosystem, the most important of which include promoting shared decision-making, implementing value-based payment models, strengthening clinical guidelines, increasing provider education, encouraging public awareness, strengthening research and innovation, and even using new methods developed with the help of artificial intelligence (8).

Shared decision-making enables patients to participate actively in their care by understanding proposed interventions' risks, benefits, and alternatives. This approach helps strengthen the doctor-patient relationship while reducing the likelihood of unnecessary treatments. The shift from fee-for-service payment to value-based payment models encourages providers to provide high-quality, cost-effective care. Such systems reward outcomes rather than volume and align financial incentives with patient needs. Evidence-based clinical guidelines can help standardize care and reduce unnecessary variations. Regular updates of these guidelines ensure that they reflect the latest scientific findings and best practices. The use of systematic reviews and health technology assessments in this area is highly encouraged. Continuing medical education programs should emphasize the risks of overuse and equip providers with strategies to navigate challenging scenarios, such as managing patient expectations and practicing responsible defensive medicine. Public awareness campaigns such as the “Choosing Wisely Campaign” can educate consumers about the risks of overuse and promote a culture of inquiry and informed consent. Investment in research is critical to identify undervalued practices and develop alternative approaches. In addition, innovations in digital health, such as artificial intelligence-based decision support systems, promise to improve diagnostic accuracy and reduce overuse (9).

Medical overuse represents a complex and global challenge that requires urgent attention and coordinated action from all sectors of society and international health organizations. By addressing the underlying drivers of overuse and implementing targeted and precise solutions, we can create a healthcare system that prioritizes patient safety, improves efficiency, and ensures sustainable resource allocation. As stakeholders in the healthcare community, it is our collective responsibility to support these changes and work toward a future in which every medical intervention truly adds value to patient care. We hope that with the development of research in this area and greater attention from policymakers, we will see a decrease in unnecessary health interventions and an increase in necessary, high-quality, and cost-effective interventions in health systems.

## References

[1] Thacharodi A Singh P Meenatchi R Tawfeeq Ahmed Z Kumar RR Kavish S. Revolutionizing healthcare and medicine: the impact of modern technologies for a healthier future—A comprehensive review. Health Care Sci. (2024) 3:329–49. 10.1002/hcs2.115 39479277 PMC11520245

[2] Pezeshki MZ Janati A Arab-Zozani M. Medical overuse in the Iranian healthcare system: a systematic scoping review and practical recommendations for decreasing medical overuse during unexpected COVID-19 pandemic opportunity. Risk Manag Healthc Policy. (2020) 13:1103–10. 10.2147/RMHP.S262908 32848487 PMC7429239

[3] Arab-Zozani M Pezeshki MZ Khodayari-Zarnaq R Janati A. Medical overuse in the Iranian healthcare system: a systematic review protocol. BMJ Open. (2018) 8:e020355. 10.1136/bmjopen-2017-020355 29666133 PMC5905767

[4] Brownlee S Chalkidou K Doust J Elshaug AG Glasziou P Heath I. Evidence for overuse of medical services around the world. Lancet. (2017) 390:156–68. 10.1016/S0140-6736(16)32585-5 28077234 PMC5708862

[5] Segal JB Sen AP Glanzberg-Krainin E Hutfless S. Factors associated with overuse of health care within US health systems: a cross-sectional analysis of Medicare beneficiaries from 2016 to 2018. JAMA Health Forum. (2022) 3:e214543. 10.1001/jamahealthforum.2021.4543 35977230 PMC8903118

[6] Albarqouni L Abukmail E MohammedAli M Elejla S Abuelazm M Shaikhkhalil H. Low-value surgical procedures in low-and middle-income countries: a systematic scoping review. JAMA Netw Open. (2023) 6:e2342215. 10.1001/jamanetworkopen.2023.42215 37934494 PMC10630901

[7] Albarqouni L Arab-Zozani M Abukmail E Greenwood H Pathirana T Clark J. Overdiagnosis and overuse of diagnostic and screening tests in low-income and middle-income countries: a scoping review. BMJ Global Health. (2022) 7:e008696. 10.1136/bmjgh-2022-008696 36316027 PMC9442491

[8] Kakemam E Arab-Zozani M Raeissi P Albelbeisi AH. The occurrence, types, reasons, and mitigation strategies of defensive medicine among physicians: a scoping review. BMC Health Serv Res. (2022) 22:800. 10.1186/s12913-022-08194-w 35725449 PMC9210603

[9] Hasanpoor E Janati A Arab-Zozani M Haghgoshayie E. Using the evidence-based medicine and evidence-based management to minimise overuse and maximise quality in healthcare: a hybrid perspective. BMJ Evidence-Based Medicine. (2020) 25:3–5. 10.1136/bmjebm-2018-110957 30355659

